# Supplementary figures and images for: Hypercontractile Cardiac Phenotype in Mice with Migraine-Associated Mutation in the Na+,K+-ATPase α2-Isoform
Source: Cells. 2023 Apr 7;12(8):1108. doi: 10.3390/cells12081108 (PMC10136638; doi:10.3390/cells12081108)

**a**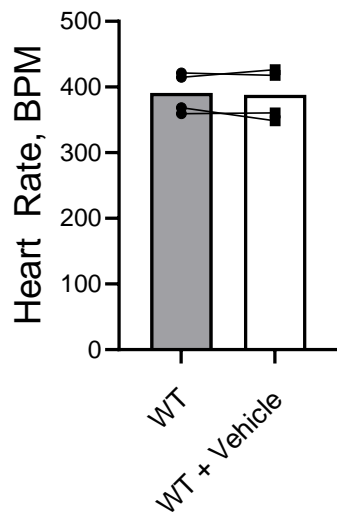**b**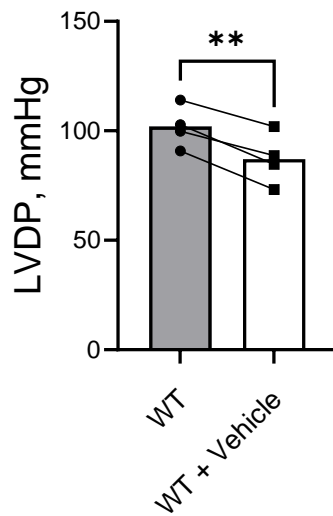**c**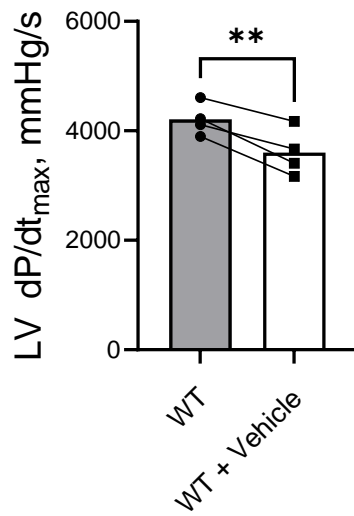**d**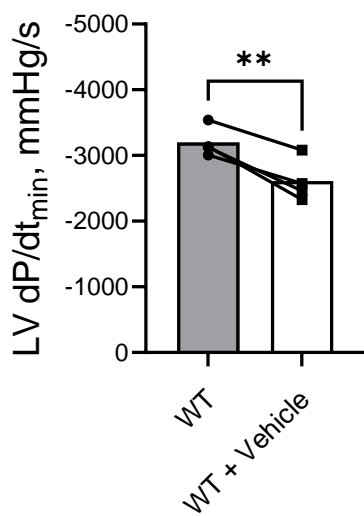**e**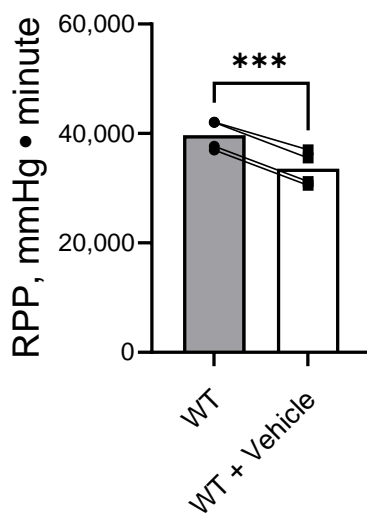**f**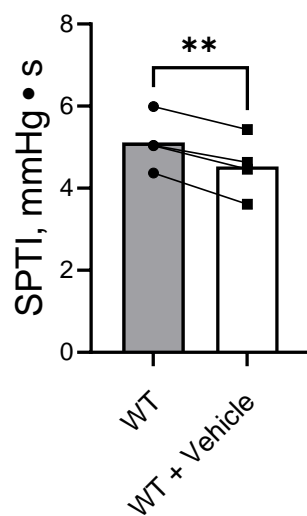**g**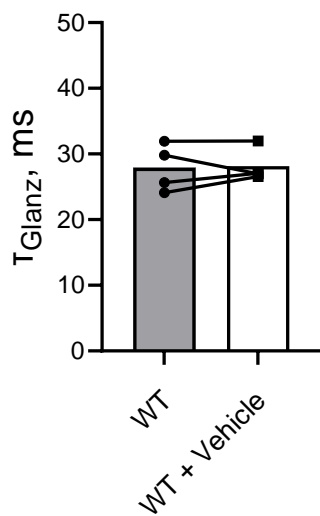**h**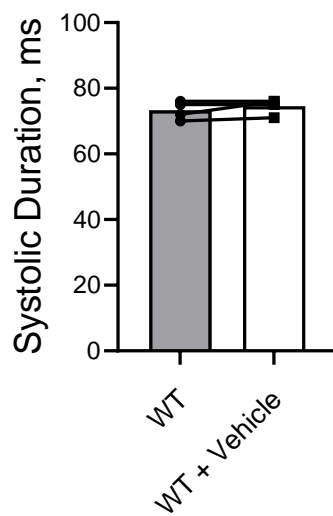

Supplement: Supplementary file 1 [file cells-12-01108-s001.zip › Revvised supplementary figures/Supplementary 1_time effect of vehicle.pdf]

**a**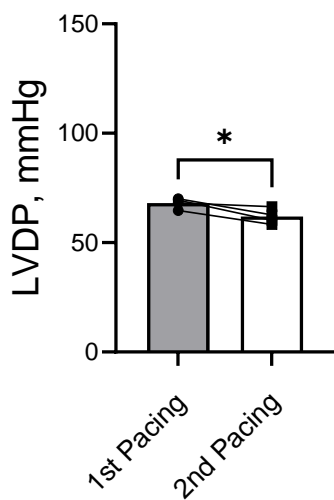**b**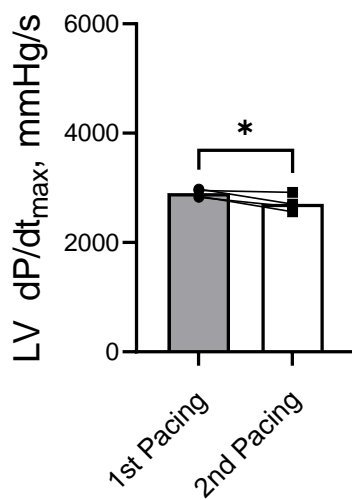**c**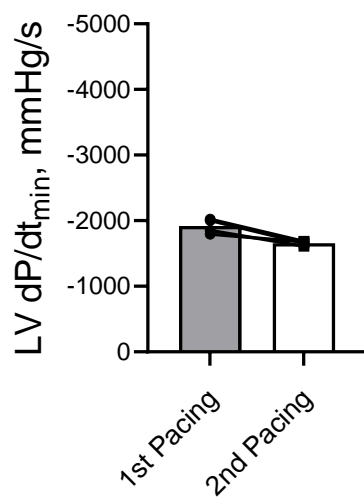**d**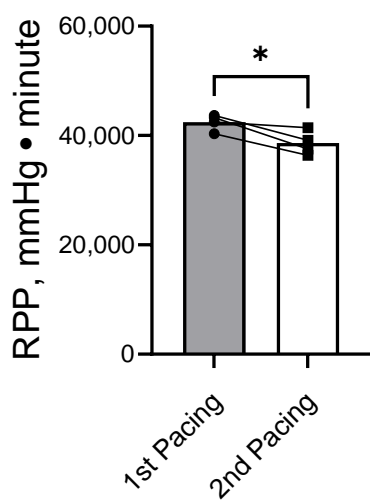**e**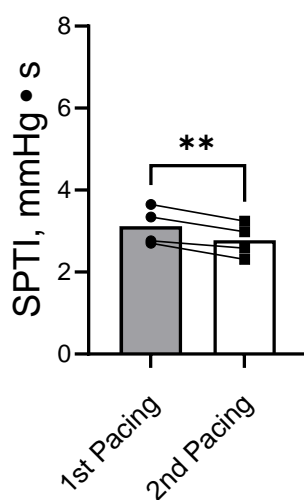**f**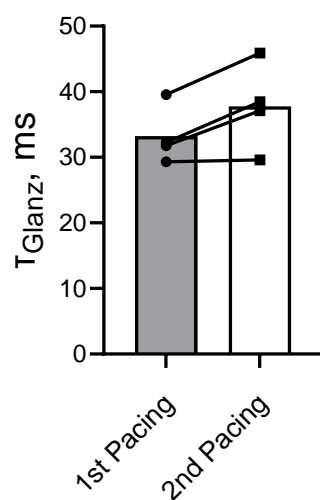**g**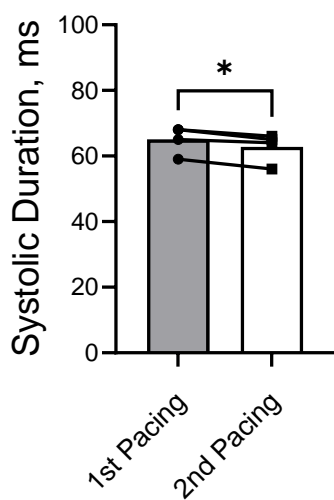

Supplement: Supplementary file 1 [file cells-12-01108-s001.zip › Revvised supplementary figures/Supplementary 2_timecontrolpacing.pdf]
